# Neurodevelopmental dimensional assessment of young children at high genomic risk of neuropsychiatric conditions

**DOI:** 10.1002/jcv2.12162

**Published:** 2023-05-04

**Authors:** Samuel J. R. A. Chawner, Amy L. Paine, Matt J. Dunn, Alice Walsh, Poppy Sloane, Megan Thomas, Alexandra Evans, Lucinda Hopkins‐Jones, Siske Struik, IMAGINE‐ID consortium, F. Lucy Raymond, Eleanor Dewhurst, Amy Lafont, Husniye Timur, Francesca Wicks, Zheng Ye, Kate Baker, Neil Walker, Sarah Wallwork, David Skuse, Spiros Denaxas, William Mandy, Jeanne Wolstencroft, Sarah Davies, Marie Erwood, Manoj Juj, Eleanor Kerry, Anna Lucock, Frida Printzlau, Ramya Srinivasan, Susan Walker, Nadia Coscini, Nasrtullah Fatih, Hayley Denyer, Sophie Andrews, Samuel Chawner, Andrew Cuthbert, Aimee Challenger, Jeremy Hall, Nicola Lewis, Michael Owen, Sinead Ray, Matthew Sopp, Hayley Moss, Marianne van den Bree, Peter Holmans, Samantha Bowen, Karen Bradley, Philippa Birch, Molly Tong, Tasmin Ford, Beverly Searle, Sarah Wynn, Lisa Robertson, Jonathan Berg, Anne Lampe, Shelagh Joss, Paul Brennan, Alison Kraus, Astrid Weber, Myfanwy Rawson, Oliver Quarrell, Pradeep Vasudevan, Rachel Harrison, Denise Williams, Eamonn Maher, Usha Kini, Virginia Clowes, Fleur Van Dijk, Jana Gurasashvilli, Sahar Mansour, Muriel Holder‐Espinasse, Amy Watford, Julia Rankin, Diana Baralle, Annie Procter, Jeremy Hall, Jonathan T. Erichsen, Susan R. Leekam, Michael J. Owen, Dale Hay, Marianne B. M. van den Bree

**Affiliations:** ^1^ Medical Research Council Centre for Neuropsychiatric Genetics and Genomics Division of Psychological Medicine and Clinical Neurosciences Cardiff University Cardiff UK; ^2^ Cardiff University Centre for Human Developmental Science School of Psychology Cardiff University Cardiff UK; ^3^ School of Optometry and Vision Sciences Cardiff University Cardiff UK; ^4^ Immunodeficiency Centre for Wales University Hospital of Wales Cardiff UK

**Keywords:** 22q11.2 Deletion Syndrome, child & adolescent mental health, genomics, neurodevelopmental conditions, transdiagnostic

## Abstract

**Background:**

Individuals with 22q11.2 deletion are at considerably increased risk of neurodevelopmental and psychiatric conditions. There have been very few studies investigating how this risk manifests in early childhood and what factors may underlie developmental variability. Insights into this can elucidate transdiagnostic markers of risk that may underlie later development of neuropsychiatric outcomes.

**Methods:**

Thirty two children with 22q11.2 Deletion Syndrome (22q11.2DS) (mean age = 4.1 [SD = 1.2] years) and 12 sibling controls (mean age = 4.1 [SD = 1.5] years) underwent in‐depth dimensional phenotyping across several developmental domains selected as being potential early indicators of neurodevelopmental and psychiatric liability. Comparisons were conducted of the dimensional developmental phenotype of 22q11.2DS and sibling controls. For autistic traits, both parents and children were phenotyped using the Social Responsiveness Scale.

**Results:**

Young children with 22q11.2DS exhibited large impairments (Hedge's *g* ≥ 0.8) across a range of developmental domains relative to sibling controls, as well as high rates of transdiagnostic neurodevelopmental and psychiatric traits. Cluster analysis revealed a subgroup of children with 22q11.2DS (*n* = 16; 53%) in whom neurodevelopmental and psychiatric liability was particularly increased and who differed from other children with 22q11.2DS and non‐carrier siblings. Exploratory analyses revealed that early motor and sleep impairments indexed liability for neurodevelopmental and psychiatric outcomes. Maternal autism trait scores were predictive of autism traits in children with 22q11.2DS (intraclass correlation coefficients = 0.47, *p* = 0.046, *n* = 31).

**Conclusions:**

Although psychiatric conditions typically emerge later in adolescence and adulthood in 22q11.2DS, our exploratory study was able to identify a range of early risk indicators. Furthermore, findings indicate the presence of a subgroup who appeared to have increased neurodevelopmental and psychiatric liability. Our findings highlight the scope for future studies of early risk mechanisms and early intervention within this high genetic risk patient group.

## Graphical abstract

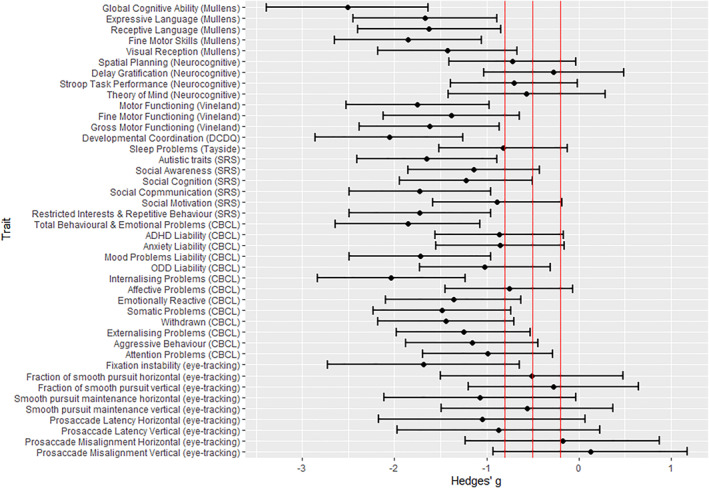

**Key points**

- 22q11.2 deletion syndrome (22q11.2DS) is a genetic condition that greatly increases liability for neurodevelopmental and psychiatric outcomes. Examining the early developmental phenotype in 22q11.2DS could elucidate early transdiagnostic markers of childhood vulnerability.
- Young children with 22q11.2DS and their unaffected siblings were assessed using a range of dimensional measures informed by the Research Domain Criteria framework; including sleep functioning, cognitive, motor, reward valuation and social processes.
- Although neuropsychiatric conditions typically emerge later in adolescence in 22q11.2DS, our study identified a range of risk indicators in early childhood, indicating scope for future studies of early risk mechanisms and early intervention in this at‐risk patient group.

## INTRODUCTION

22q11.2 Deletion Syndrome (22q11.2DS) has been identified as one of the strongest risk factors for the development of neurodevelopmental and psychiatric conditions across the lifespan, including intellectual disability, attention deficit hyperactivity disorder (ADHD), anxiety and mood disorders, autism spectrum conditions, and is one of strongest known biological risk factors for schizophrenia (Malhotra & Sebat, 2012; Murphy et al., 1999; Niarchou et al., 2014; Rees et al., 2016; Sanders et al., 2015; Schneider et al., 2014). Clinical studies report over 60% of individuals who have been diagnosed with 22q11.2DS in a medical genetic clinic setting meet criteria for a psychiatric condition (Bertrán et al., 2018; Jonas et al., 2014). 22q11.2DS has also been associated with psychiatric risk in genome‐wide association studies (Malhotra & Sebat, 2012; Rees et al., 2014), and within population cohorts (Olsen et al., 2018). Although the effect size of associations between 22q11.2DS and psychiatric outcomes differs by study design and ascertainment strategy (Fiksinski, Schneider, et al., 2021), there is an overarching consensus across studies that it is associated with elevated psychiatric risk.

22q11.2DS affects 1 in 4000 live births and is considered the most frequent chromosomal microdeletion syndrome (McDonald‐McGinn et al., 2015), yet there is still relatively low awareness of this condition in the clinical community. 22q11.2DS is typically diagnosed within the first years of life, usually following referral to medical genetics clinics for a range of clinical features, particularly congenital abnormalities and developmental concerns (Cancrini et al., 2014). This provides unique opportunities for prospective deep phenotyping to identify transdiagnostic markers of risk (Baker & Vorstman, 2012) that manifest early in development and underlie later development of psychiatric conditions. This knowledge is needed to develop tailored services for prevention and early detection and management of serious mental illness.

Most studies of 22q11.2DS in childhood have focused on children aged 6 years and above, and these have established that divergent cognitive and psychopathology trajectories are present in late childhood and adolescence (Chawner et al., 2017; Chawner, Niarchou, et al., 2019; Morrison et al., 2020; Schneider et al., 2014; Tang & Gur, 2018). Few studies of psychopathology in children with 22q11.2DS have focused on earlier developmental periods. We are only aware of only two such studies (Klaassen et al., 2013; Kortanek et al., 2022). In this, 1.5 to 6‐year‐olds with 22q11.2DS were screened with the Child Behaviour Checklist (CBCL) to investigate the presence of early behavioural and emotional problems (Klaassen et al., 2013), and it was found that 30% of children met clinical cut‐offs for behavioural and emotional problems. Three studies have described a high prevalence of developmental concerns in preschool children with 22q11.2DS (Gerdes et al., 1999, 2001; Kortanek et al., 2022). 54% of children with 22q11.2DS have significant developmental delay, and 80% show delays in language development (Gerdes et al., 1999, 2001). Although previous work highlights atypical early development, the phenotypic depth of these studies has been limited, and there is a need for further studies with wide‐ranging assessments to capture a breadth of early indicators of neurodevelopmental and psychiatric liability. Furthermore, previous studies of early childhood in 22q11.2DS have not consistently included a group of unaffected controls to investigate the extent to which developmental features in 22q11.2DS are specific. Therefore, the impact of 22q11.2DS on early development relative to typically developing children has not been quantified.

An additional limitation of previous work has been a focus on categorical clinical cut‐offs, rather than the dimensions that underlie early childhood impairment. The National Institute of Mental Health has developed a framework (Research Domain Criteria [RdoC]) which—rather than characterising individuals in terms of categorical diagnostic mental health disorders—uses a broader approach based on dimensional constructs that underlie these conditions. These RdoC framework dimensional measures are not simply continuous measures of categorical psychiatric diagnoses; instead, there is a focus on measures that index the underlying mechanisms and neurobiological bases of psychiatric conditions. Methodologies for assessing potential dimensional RdoC traits range from subjective questionnaire measures to objective assessment of traits via approaches including cognitive testing and experimental eye‐tracking paradigms (National Institute of Mental Health, 2011). Previous studies of school‐age children with 22q11.2DS have reported behavioural (Schneider et al., 2014), cognitive (Fiksinski, Bearden, et al., 2021) and motor impairments (Cunningham et al., 2018), but the extent to which these covary in early development remains unclear. Intellectual impairments in school‐age children with 22q11.2DS have been found to be independent of the presence of childhood neurodevelopmental and psychiatric diagnoses, including ADHD, autism, and anxiety (Niarchou et al., 2014), but the extent this applies to earlier developmental periods has not been investigated.

The RDoC approach offers increased opportunities to examine transdiagnostic liability (Doherty & Owen, 2014; Insel et al., 2010). This approach may be particularly informative in young children, as it can capture subthreshold liability not yet manifested as a clinical diagnosis but that may be indicative of later risk. Another benefit of dimensional measures is that they open the possibility of investigating whether subthreshold traits in relatives predict outcomes in children at risk. Variability in cognitive and psychiatric traits in 22q11.2DS has been found to be predictable, based on the cognitive and psychiatric profiles of relatives (Klaassen et al., 2014; Olszewski et al., 2014), highlighting the fact that factors beyond the deletion influence neurodevelopmental and psychiatric outcomes. However, the extent to which this applies to early development in 22q11.2DS has not previously been investigated, and therefore it is unclear whether phenotypic correlations between children and relatives emerge through development or if this is present from early development.

Here we present findings from young children aged 2–5 years old with 22q11.2DS compared to unaffected sibling controls. Children completed a broad phenotyping battery which included: (a) age appropriate dimensional measures (Hay et al., 2021) aligned to the RdoC framework, and (b) measures of neurodevelopmental and psychiatric liability from which both dimensional and categorical outcomes could be derived. Our specific aims were: (1) to assess the early developmental profiles of children with 22q11.2DS relative to controls; (2) to investigate whether cognitive, motor and sleep traits were associated with neurodevelopmental and psychiatric liability, and (3) to investigate whether variability in early childhood outcomes is influenced by the phenotypes of relatives.

## METHODOLOGY

Children with 22q11.2DS were identified using existing partnerships developed as part of the ECHO (Chawner et al., 2017; Niarchou et al., 2014) and IMAGINE‐ID studies (Chawner, Owen, et al., 2019), including via NHS medical genetics clinics, the patient support group Max Appeal, and social media. Children were eligible if they were aged between 24 and 71 months and had a diagnosis of 22q11.2DS confirmed by a medical genetics clinic; control siblings without 22q11.2DS within the same age range were also invited to take part. Exclusion criteria based on neurodevelopmental screening was not applied to either group, including the sibling controls. Thirty‐two children with 22q11.2DS (mean = 4.1 years [SD = 1.2]; 34% male) took part, as well as 12 sibling controls (mean = 4.1 years [SD = 1.5]; 50% male). The 22q11.2DS and control sibling groups did not significantly differ in age (two‐sample *t*‐test, *p* = 0.995). Presence of a deletion at the 22q11.2DS locus was confirmed via medical records and by micro‐array analysis in the Cardiff University Division of Psychological Medicine and Clinical Neurosciences laboratory. Absence of the deletion in control siblings was established by microarray analysis in the same laboratory. Nine per cent (3/32) of children with 22q11.2DS and 17% (2/12) of sibling controls had non‐European ancestry. Information regarding maternal education and household income as reported by the primary caregiver is available in Table 1. Regarding psychotropic or neurological related medications, one child was currently taking a GABA agonist for dystonia, and two children were taking melatonin for sleep problems.

**TABLE 1:** Demographics of children with 22q11.2DS and controls.

	22q11.2DS	Controls
Maternal education		
University degree and/or other higher postgraduate qualification	50% (16/32)	42% (5/12)
A‐levels/highers or vocational training	13% (4/32)	42% (5/12)
O‐levels or GCSEs	34% (11/32)	17% (2/12)
Unknown	3% (1/32)	0% (0/12)
Household income		
<£20,000	16% (5/32)	25% (3/12)
£20,000—£39,999	28% (9/32)	33% (4/12)
£40,000—£59,999	28% (9/32)	42% (5/12)
≥£60,000	19% (6/32)	0% (0/12)
Unknown/not disclosed	9% (3/32)	0% (0/12)

### Assessments

Parents and caregivers were asked to complete questionnaires in advance of the research assessment appointment, and interviews took place at the assessment. Children with 22q11.2DS and sibling controls within the same family were assessed on the same day, but in separate rooms by separate raters.

### Developmental history

Developmental history was ascertained via caregiver report questionnaires and interviews.

#### Developmental milestones

The developmental milestones section of the *Autism Diagnostic Interview—Revised (ADI‐R)* (Rutter et al., 2003) was administered with the caregiver to ascertain the age in months in which milestones were achieved for walking, toilet training, and language development.

#### Neurological health

The Epilepsy Screening Questionnaire (ESQ) (Ottman et al., 2010) was administered to screen for epilepsy diagnosis and seizure‐like symptoms.

### Dimensional phenotyping

Table 2 provides an overview of the range of dimensional phenotypic traits that were assessed and how they align to RDoC domains and psychiatric phenomenology.

**TABLE 2:** Overview of dimensional traits assessed.

Trait	Measure	System	Construct	Framework
Sleep problems	Tayside questionnaire	Arousal and regulatory systems	Sleep	RDoC
Global cognitive ability	Mullens	Cognitive systems	‐	
Expressive language	Mullens		Language	
Receptive language	Mullens		Language	
Visual reception	Mullens		Visual perception	
Spatial planning	Neurocognitive battery		Cognitive control	
Stroop task performance (neurocognitive)	Neurocognitive battery		Cognitive control	
Fine motor skills	Mullens	Motor	‐	
Motor functioning	Vineland			
Fine motor functioning	Vineland			
Gross motor functioning	Vineland			
Developmental coordination	DCDQ			
Smooth pursuit eye movements	Eye‐tracking			
Prosaccade eye movements	Eye‐tracking			
Fixation instability	Eye‐tracking			
Delay gratification	Neurocognitive battery	Positive & negative valence systems	Reward valuation	
Theory of mind	Neurocognitive battery	Social processes	Theory of mind	
Social awareness	Social responsiveness scale		Social functioning	
Social cognition	Social responsiveness scale		Social functioning	
Social communication	Social responsiveness scale		Social functioning	
Social motivation	Social responsiveness scale		Social functioning	

Trait	Measure	Type of measure	Framework
Total behavioural & emotional problems (CBCL)	CBCL	Overall neuropsychiatric liability	Phenomenology
ADHD liability (CBCL)	CBCL	Diagnostic category based	
Anxiety liability (CBCL)	CBCL		
Mood problems liability (CBCL)	CBCL		
ODD liability (CBCL)	CBCL		
Autistic traits	Social responsiveness scale		
Internalising problems (CBCL)	CBCL	Transdiagnostic	
Affective problems (CBCL)	CBCL		
Emotionally reactive (CBCL)	CBCL		
Somatic problems (CBCL)	CBCL		
Withdrawn (CBCL)	CBCL		
Externalising problems (CBCL)	CBCL		
Aggressive behaviour (CBCL)	CBCL		
Attention problems (CBCL)	CBCL		
Restricted interests & repetitive behaviour (SRS)	Social responsiveness scale		

*Note*: Global Cognitive Ability refers to Mullen's *Early Learning Composite* score. Full details and references for the measures listed can be found in the methodology section.

### Cognitive function

All children completed the Mullen Scales of Early Learning (Mullen, 1995). The assessment does not rely on understanding of verbal instructions, therefore maximising accessibility of the assessment. From the assessment, the following scores were derived: *Early Learning Composite* (akin to a global intellectual score), *Visual Reception* domain score, *Fine Motor* domain score, *Expressive Language* domain score, and *Receptive Language* domain score.

To capture broader neurocognitive functioning, we administered developmentally appropriate tasks from the Cardiff Child Development Study (Hay et al., 2021), including the *Tower of Cardiff* planning task, *delay of gratification* task, *Big Bear, Little Be*ar nonverbal Stroop task. Full details of these tasks are published elsewhere (Meeuwsen et al., 2019). Theory of Mind was assessed using tasks from Wellman and Liu's (2004) theory of mind scale (Wellman & Liu, 2004).

### Oculomotor function

Children completed established eye‐tracking paradigms to assess voluntary and spontaneous oculomotor behaviour, including tests of *prosaccades, smooth pursuit* and *fixation* (Barton et al., 2008; Holzman, 2000; Morita et al., 2020). Full details of eye‐tracking methodology can be found in the Supporting Information S1.

### Neurodevelopmental and psychiatric liability

The Preschool CBCL (Achenbach, 2001) caregiver report questionnaire was administered from which the following scores were derived: (a) transdiagnostic domains including *Internalising Problems* (comprised of *Emotionally Reactive*, *Anxious/Depressed*, *Somatic Complaints*, *Withdrawn* domain scores), *Externalising Problems* (comprised of *Attention Problems*, and *Aggressive Behaviour* domain scores), and *Stress Problems*; (b) DSM‐oriented scales including liability scales for *Depressive Problems*, *Anxiety Problems*, *Attention Deficit/Hyperactivity Problems*, and *Oppositional Defiant Problems*. *Total Problems* Score was also derived which represented overall neurodevelopmental and psychiatric liability.

The Social Responsiveness Scale Second Edition (SRS‐2) (Constantino & Gruber, 2012) provides a dimensional measure of autism‐related traits. The caregiver report version of the SRS was administered to ascertain autism traits in children. The following standardised scores were derived: SRS Total *T*‐Score, Social Awareness *T*‐score, Social Cognition *T*‐score, Social Communication *T*‐score, Social Motivation *T*‐score, RRB (Restricted Interests and Repetitive Behaviour) *T*‐Score. The adult report version of the SRS was administered to ascertain autism traits in biological mothers and fathers.

### Motor development

We used two measures of motor functioning: (a) The Preschool Developmental Coordination Questionnaire (Little DCDQ) (Rihtman et al., 2011), was used to screen for liability for Developmental Coordination Disorder (Wilson et al., 2000); (b) The motor sections of the Vineland Adaptive Behaviour Scales (Sparrow et al., 1984) (designed for children with intellectual disabilities) were used to provide an assessment of motor functioning, from which standardised scores for Overall *Motor Functioning*, *Gross Motor* Functioning, and *Fine Motor* Functioning were derived.

### Sleep functioning

The Tayside Children's Sleep Questionnaire (TCSQ) was used to screen for disorders of initiating and maintaining sleep in children aged between 1 and 5 years (McGreavey et al., 2005). A symptom score can be derived from the TCSQ, as well as a categorical indicator of clinically relevant sleep problems.

### Statistical analysis

#### Comparing developmental history between 22q11.2DS and controls

For each developmental milestone, as ascertained from the developmental interview, age was compared between children with 22q11.2DS and controls using linear mixed models that controlled for gender and age as fixed effects, and familial relatedness as a random effect (to account for the fact that some children with 22q11.2DS and controls came from the same family). Missing variables occurred as not all caregivers were able to retrospectively provide exact age in months for developmental milestones. For analysis of epilepsy‐related variables, we conducted Fisher's exact tests for seizure presence due to 0 cell count for the controls. For analysis of presence of epilepsy symptoms, mixed effect models for binary outcomes failed to converge, so we opted for logistic regression models with gender and age as covariates.

#### Contrasting 22q11.2DS with controls on dimensional and categorical measures of cognition, motor development, sleep function and neurodevelopmental and psychiatric liability

For each dimensional trait, z‐scores were calculated for children with 22q11.2DS relative to the sibling controls (reference group), which were adjusted for the covariates of gender and age. Scores were constructed so that a negative score indicated atypical development. Linear mixed models were conducted to investigate developmental differences between children with 22q11.2DS and controls, whereby gender and age were fixed effects and familial relatedness was included as random effect. Benjamini‐Hochberg false discovery rate (B‐H FDR) multiple testing correction value of 0.05 was applied to analyses. To examine the effect size of developmental differences, Hedges' *g* values were calculated using dimensional trait scores adjusted for gender and age. Hedges' *g* takes account of the difference in sample size between sibling controls and 22q11.2DS groups. Age‐matched population norm data was available for the majority of traits assessed (Mullen Scales of Early Learning, CBCL, Vineland, and SRS‐2) (Achenbach, 2001; Constantino & Gruber, 2012; Mullen, 1995; Sparrow, 2011), whilst for others where possible we were able to use previously collected community control data (Tayside questionnaire, DCDQ, Cardiff Child Development Study tasks, Theory of Mind scale) (Hay et al., 2014, 2021; McGreavey et al., 2005; Meeuwsen et al., 2019; Rihtman et al., 2011; Wellman & Liu, 2004). Population norm data or community control data was not available for the eye‐tracking tasks. *Z*‐tests were conducted to investigate how the 22q11.2DS and sibling control groups performed relative to samples representative of the general population. Correlations between the dimensional traits were calculated to explore the relationships between traits. Exploratory K‐means cluster analysis was conducted to categorise children with 22q11.2DS and controls into clusters based on phenotypic similarity. Previous neurodevelopmental research with a similar sample size has used exploratory cluster analysis to characterise transdiagnostic heterogeneity (McDougal et al., 2020). The following dimensional traits, adjusted for age and gender, were included in the cluster analysis: from the Mullen Scales of Early Learning (cognition); CBCL (neurodevelopmental and psychiatric traits; DSM‐based and transdiagnostic), SRS (autistic traits), Vineland (motor functioning), DCDQ (motor coordination) and the Tayside questionnaire (sleep). The following summary scores were excluded from the cluster analysis due to being mathematically related to their subdomain scores which were already included in the analysis: SRS Total Score, Total CBCL Score, Internalising Problems CBCL Score, Externalising Problems CBCL Sore, Vineland Motor Standard Score and the Mullen General Cognitive Ability Score. Dimensional traits derived from the eye‐tracking tasks were not included in the cluster analysis due to missing data, as not all children engaged in the task and not all data passed strict quality control thresholds (see Table S1). The *fviz_nbclust* R package was used to identify the optimal number of clusters.

Children with 22q11.2DS and controls were also compared on categorical variables based on established clinical screening thresholds, including Global Cognitive Ability (Mullen *Early Learning Composite* score ≤85 indicates below average ability), Vineland Motor functioning impairment (standardised score ≤85), Developmental Coordination liability (≤67 for boys, ≤68 for girls), SRS (standardised score ≥60), Total CBCL score and DSM (Anxiety, ADHD, Mood and ODD) subscales (clinical liability categories derived by CBCL software based on score, age and gender), and sleep problems (clinical threshold ≥8). When comparing prevalence of these clinical liability indicators between 22q11.2DS and controls, mixed effect models taking account of fixed effects of age, gender and relatedness as a random effect were conducted. For variables where the model failed to converge, logistic regression models were constructed with age and gender as covariates. For some categorical variables there was 0 cell count for controls, and thus Fisher's exact test was used to compare prevalence between groups where logistic regression was not appropriate. B‐H FDR multiple testing correction value of 0.05 was applied to analyses.

#### Association of cognitive, motor and sleep markers with neurodevelopmental and psychiatric outcomes in children with 22q11.2DS

Exploratory correlation analyses were conducted to investigate which cognitive and motor traits indexed neurodevelopmental and psychiatric outcomes, including autistic traits (SRS) and total score for Behavioural and Emotional Problems (CBCL). Trait scores were standardised into z‐scores adjusted for age and gender before being included in correlation analyses. B‐H FDR multiple testing correction value of 0.05 was applied to analyses.

#### Influence of familial traits on childhood traits

We investigated if the SRS score of children with 22q11.2DS was influenced by biological parental SRS score, following the approach of a previous study (Moreno‐De‐Luca et al., 2015) that calculated intraclass correlation coefficients (ICC). Here, SRS ICC scores were calculated for 31 mother‐child pairs and 22 father‐child pairs.

## RESULTS

### Comparing developmental history between 22q11.2DS and controls

The following results were based on the parent report developmental history interview. For learning to walk independently, children with 22q11.2DS on average developed this skill 8 months later than their siblings (22q11.2DS mean age [months] = 20.0, SD = 5.7, *n* = 29; control mean age [months] = 11.8, SD = 3.0, *n* = 10; *p* < 0.001). All children in both groups had achieved the milestone of first spoken word, however children with 22q11.2DS were on average delayed by 14 months compared to their siblings (22q11.2DS mean age [months] = 26.7, SD = 9.9, *n* = 27; control mean age [months] = 12.4, SD = 3.4, *n* = 10; *p* < 0.001). Eighty‐five per cent (23/27) of children with 22q11.2DS had developed phrase speech by the time of assessment, whereas all controls had developed this (10/10). Amongst those who had developed phrase speech, children with 22q11.2DS were on average delayed by 16 months compared to siblings (22q11.2DS mean age [months] = 35.1, SD = 13.5, *n* = 23; control mean age [months] = 19.1, SD = 5.8, *n* = 10; *p* < 0.001).

Based on the ESQ, the presence of seizures (not including febrile) was identified in 19% (6/31) of children with 22q11.2DS, whereas none of the controls (0/12) met seizure criteria, but this difference was not statistically significant (*p* = 0.146). For febrile seizures, 3/31 children with 22q11.2DS screened positive, whereas none of the controls (0/12) met febrile seizure criteria, (*p* = 0.548). Only one child with 22q11.2DS had received a formal diagnosis of epilepsy. For the presence of broader epilepsy‐related symptomatology (any endorsed symptom on the ESQ), 55% (17/31) of children with 22q11.2DS displayed a symptom, compared with 8% (1/12) of controls (*p* = 0.008).

### Contrasting 22q11.2DS and controls on dimensional and categorical measures of cognition, motor development, sleep function, and neurodevelopmental and psychiatric liability

**FIGURE 1:**
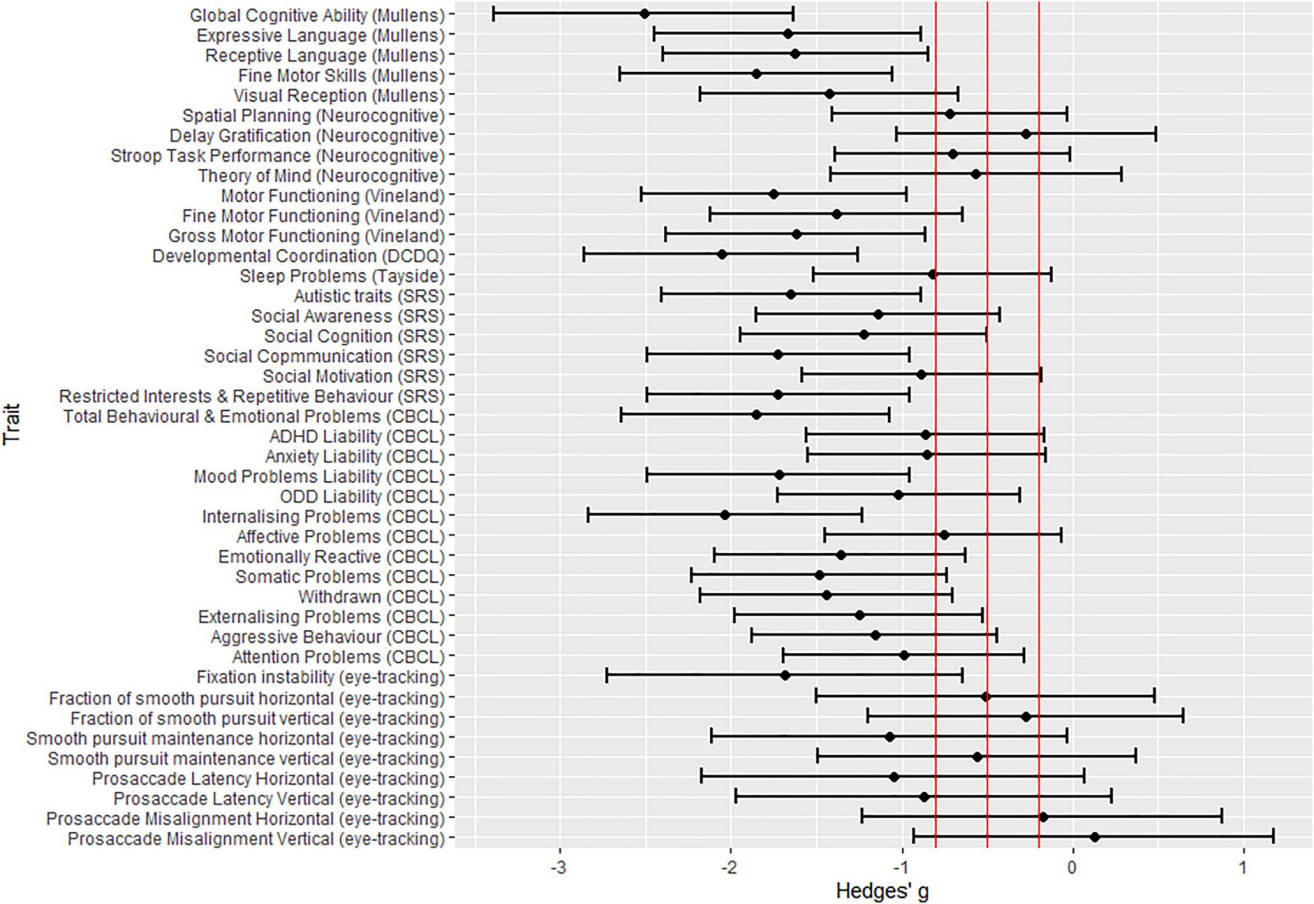
Standardised difference between 22q11.2DS and controls on dimensional cognitive and behavioural traits. Hedges' *g* represents the standardised difference in trait score between 22q11.2DS and controls, adjusted for age and gender. The red lines denote the cut‐off of effect size descriptor categories: ≤0.19 negligible; 0.20–0.49 small; 0.50–0.79 medium; ≥0.80 large.

**FIGURE 2:**
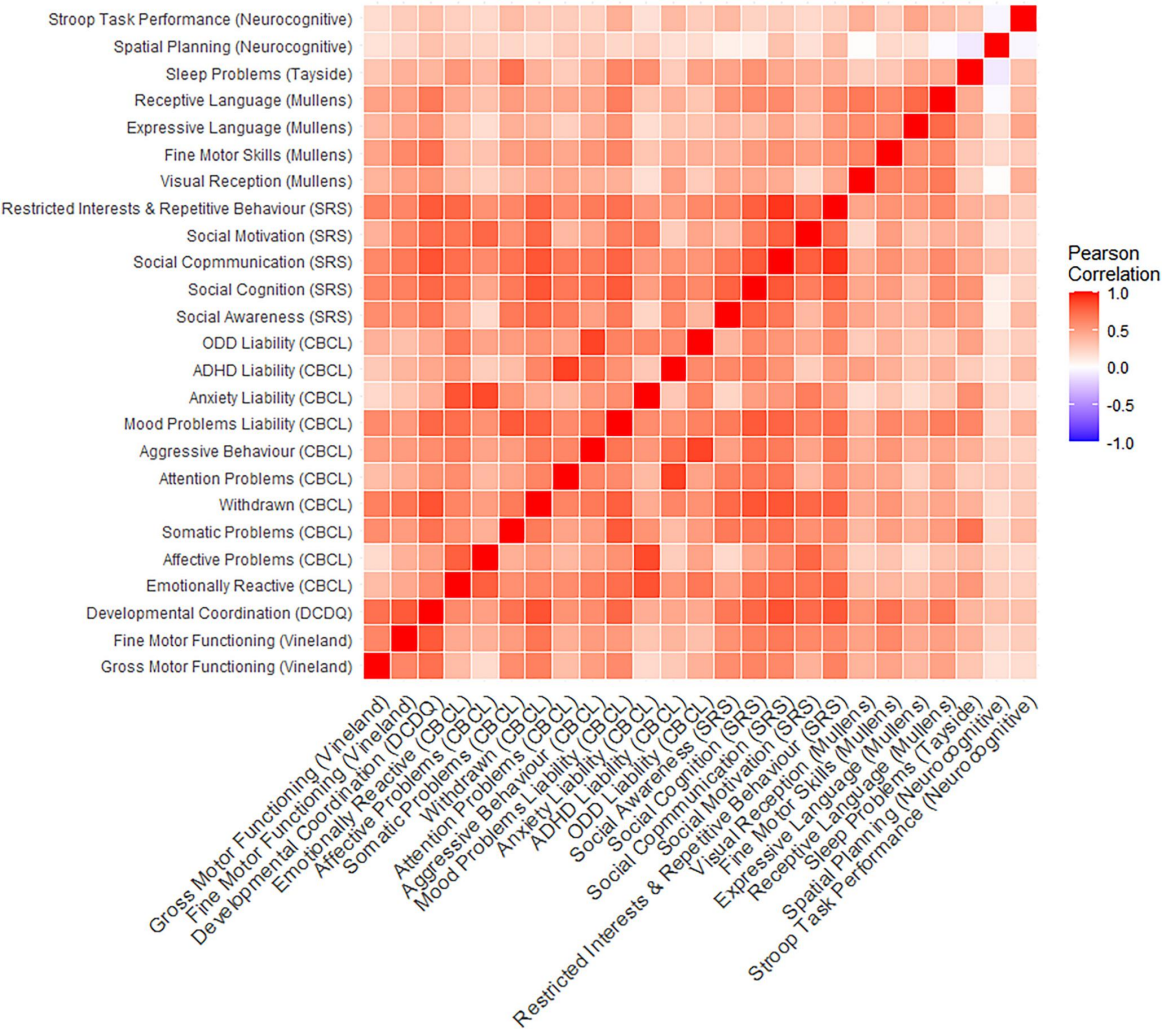
Correlation heatmap of dimensional cognitive and behavioural traits. CBCL, Child Behaviour Checklist; Little DCDQ, The Preschool Developmental Coordination Questionnaire; SRS, Social Responsiveness Scale Second Edition; TCSQ, The Tayside Children's Sleep Questionnaire.

**FIGURE 3:**
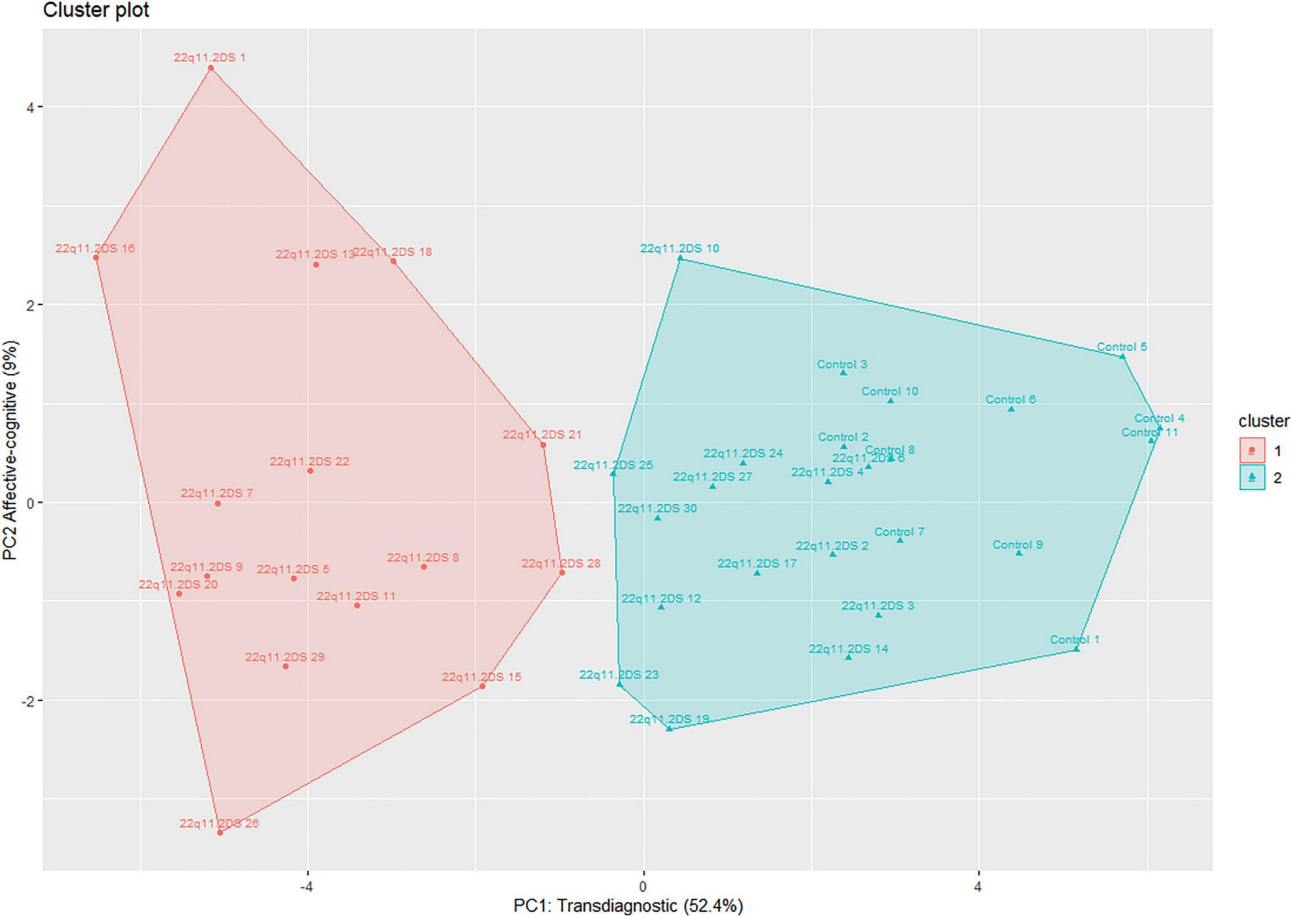
Study participants clustered based on dimensional phenotypes. Dimension 1 (Transdiagnostic) and 2 (Affective‐cognitive), represent the principal components that explained the most variability across the dimensional phenotypes.

Children with 22q11.2DS differed from controls on a range of cognitive, motor, oculomotor, sleep and neurodevelopmental and psychiatric traits analysed (Table 3; Figure 1), and the effect size of the differences (based on Hedges' *g*; Cohen, 1988) was large (≥0.8) for the majority of traits. Furthermore, the majority survived B‐H FDR multiple testing correction, and remained significant in sensitivity analyses including household income as a covariate (Table S2). Also for traits where population norm or community control data were available, children with 22q11.2DS scored significantly below the norm for the majority (28/30) of traits (Table S3, *z*‐tests *p* < 0.001). For the majority (26/30) of traits the sibling controls did not differ significantly from population norms or community control data (Table S3). Although this indicates that sibling controls were broadly representative of population norms for traits including global cognitive ability, overall autistic traits and total score for behavioural and emotional problems (CBCL), it should be noted sibling controls did score significantly higher (higher score should be interpreted as meaning experienced less problems) than population norms on four subdomain traits; social communication, internalising problems, spatial planning and Stroop task performance. The findings for spatial planning and Stroop task performance should be interpreted cautiously as only community control data was available, and not age matched normative data (see Supporting Information S1 for full discussion). The majority of correlations between different dimensional traits were positive, potentially indicating that different traits may be indexing an underlying transdiagnostic construct (see Figure 2 and Table S4). Cluster analysis of dimensional traits (full list in methodology section) identified two groups of participants (Cluster 1 = 25 participants, controls = 11, 22q11.2DS = 14; Cluster 2 = 16 participants, all children with 22q11.2DS), which accounted for 39.5% of the variance in phenotypic outcomes. Gap statistic analysis (Tibshirani et al., 2001) confirmed that two clusters provided the most optimum solution (see Figure S1). To visualise the cluster analysis, Figure 3 shows a plot of the two principal components (PC) dimensions (PC1, Transdiagnostic; PC2, Affective‐Cognitive; see Table S5 for loadings and interpretation of the PCA dimensions) that explain the most phenotypic variance and shows where each child with 22q11.2DS and controls fall within this phenotypic space. It should be noted that the purpose of the principal component plot was to visualise the cluster analysis findings, future larger studies are needed to investigate the underlying factor structure of early childhood cognitive and behavioural traits in 22q11.2DS. Inspection of the cluster group mean scores (not shown) revealed that Cluster 1 participants had less impairment on all phenotypic traits compared to Cluster 2 participants, indicating Cluster 2 participants had phenotypic scores indicative of *higher vulnerability* relative to Cluster 1 which represented *lower vulnerability*. Cluster 2 (*higher vulnerability*) consisted solely of children with 22q11.2DS, whereas Cluster 1 (*lower vulnerability*) included controls and children with 22q11.2DS. There was no evidence for significant differences in age (two sample *t*‐test, *p* = 0.352) and gender (Fisher's exact, *p* = 0.118) between children with 22q11.2DS in Cluster 1 (22q11.2DS mean age = 3.9 years, 2/14 male) and Cluster 2 (22q11.2DS mean age = 4.3, 7/16 male) though it should be noted that Cluster 1 had a higher proportion of girls with 22q11.2DS.

**TABLE 3:** Dimensional phenotype *z*‐scores for controls and 22q11.2DS and group contrasts.

Dimensional phenotypic trait	Control			22q11.2DS			Group contrasts	
	*n*	*z*‐score	SD	*n*	z‐score	SD	Hedges'g	*p*‐Value
Global cognitive ability (Mullens)	11	0.0	1	32	−2.6	1.0	−2.5	**<0.001**
Expressive language (Mullens)	11	0.0	1	32	−1.8	1.1	−1.7	**<0.001**
Receptive language (Mullens)	11	0.0	1	32	−1.7	1.0	−1.6	**<0.001**
Fine motor skills (Mullens)	11	0.0	1	32	−1.8	0.9	−1.9	**<0.001**
Visual reception (Mullens)	11	0.0	1	32	−1.7	1.2	−1.4	**<0.001**
Spatial planning (neurocognitive)	12	0.0	1	32	−1.3	2.0	−0.7	0.043
Delay gratification (neurocognitive)	9	0.0	1	29	−0.2	0.8	−0.3	0.480
Stroop task performance (neurocognitive)	12	0.0	1	32	−0.8	1.1	−0.7	**0.026**
Theory of mind (neurocognitive)	8	0.0	1	19	−0.5	0.8	−0.6	0.198
Motor functioning (Vineland)	12	0.0	1	30	−1.6	0.8	−1.8	**<0.001**
Fine motor functioning (Vineland)	12	0.0	1	30	−1.4	1.0	−1.4	**<0.001**
Gross motor functioning (Vineland)	12	0.0	1	31	−1.3	0.6	−1.6	**<0.001**
Developmental coordination (DCDQ)	12	0.0	1	31	−3.6	1.9	−2.1	**<0.001**
Sleep problems (Tayside)	12	0.0	1	31	−1.7	2.4	−0.8	**0.020**
Autistic traits (SRS)	12	0.0	1	31	−2.9	1.9	−1.6	**<0.001**
Social awareness (SRS)	12	0.0	1	31	−2.0	1.9	−1.1	**0.002**
Social cognition (SRS)	12	0.0	1	31	−1.7	1.5	−1.2	**0.001**
Social communication (SRS)	12	0.0	1	31	−3.5	2.3	−1.7	**<0.001**
Social motivation (SRS)	12	0.0	1	31	−0.8	0.9	−0.9	**0.013**
Restricted interests & repetitive behaviour (SRS)	12	0.0	1	31	−3.0	1.9	−1.7	**<0.001**
Total behavioural & emotional problems (CBCL)	12	0.0	1	31	−2.2	1.2	−1.9	**<0.001**
ADHD liability (CBCL)	12	0.0	1	31	−1.4	1.8	−0.9	**0.015**
Anxiety liability (CBCL)	12	0.0	1	31	−1.5	1.9	−0.9	**0.016**
Mood problems liability (CBCL)	12	0.0	1	31	−3.4	2.2	−1.7	**<0.001**
ODD liability (CBCL)	12	0.0	1	31	−2.0	2.2	−1.0	**0.004**
Internalising problems (CBCL)	12	0.0	1	31	−2.6	1.4	−2.0	**<0.001**
Affective problems (CBCL)	12	0.0	1	31	−1.0	1.3	−0.8	**0.031**
Emotionally reactive (CBCL)	12	0.0	1	31	−2.6	2.1	−1.4	**<0.001**
Somatic problems (CBCL)	12	0.0	1	31	−7.8	6.0	−1.5	**<0.001**
Withdrawn (CBCL)	12	0.0	1	31	−3.3	2.6	−1.4	**<0.001**
Externalising problems (CBCL)	12	0.0	1	31	−1.3	1.0	−1.3	**0.001**
Aggressive behaviour (CBCL)	12	0.0	1	31	−1.9	1.8	−1.2	**0.001**
Attention problems (CBCL)	12	0.0	1	31	−1.4	1.6	−1.0	**0.006**
Fixation stability (eye‐tracking)	8	0.0	1	13	−2.6	2	−1.7	**0.002**
Fraction of smooth pursuit horizontal (eye‐tracking)	7	0.0	1	11	−0.4	1	−0.5	0.304
Fraction of smooth pursuit vertical (eye‐tracking)	7	0.0	1	15	−0.4	2	−0.3	0.543
Smooth pursuit maintenance horizontal (eye‐tracking)	7	0.0	1	11	−1.6	2	−1.1	**0.035**
Smooth pursuit maintenance vertical (eye‐tracking)	7	0.0	1	15	−0.7	1	−0.6	0.219
Prosaccade latency horizontal (eye‐tracking)	7	0.0	1	8	−1.0	1	−1.0	0.067
Prosaccade latency vertical (eye‐tracking)	7	0.0	1	8	−0.7	1	−0.9	0.118
Prosaccade misalignment horizontal (eye‐tracking)	7	0.0	1	8	−0.2	1	−0.2	0.467
Prosaccade misalignment vertical (eye‐tracking)	7	0.0	1	8	0.2	2	0.1	0.097

*Note*: Bold numbers indicate the *p*‐value survives B‐H FDR 0.05 correction for multiple testing.

For categorical variables, children with 22q11.2DS were more likely than controls to meet criteria for cognitive impairment (22q11.2DS = 84% vs. controls = 9%; *p* = 0.001), developmental coordination disorder (22q11.2DS = 97% vs. controls = 25%; *p* < 0.001), motor functioning impairment (22q11.2DS = 84% vs. controls = 9%, *p* = 0.003), neurodevelopmental and psychiatric liability (22q11.2DS = 55% vs. controls = 0%; *p* = 0.001), mood disorder liability (22q11.2DS = 45% vs. controls = 0%; *p* = 0.003), and autism liability (22q11.2DS = 84% vs. controls = 9%; *p* = 0.003) (see Table 4). The significance of these findings survived B‐H FDR 0.05 correction for multiple testing.

**TABLE 4:** Categorical outcomes in children with 22q11.2DS and controls.

Categorical measure	Control % (*n*)	22q11.2DS % (*n*)	*p*‐Value
Cognitive impairment (Mullens)	9% (1)	84% (27)	**0.001**
Developmental coordination disorder liability (DCDQ)	25% (3)	97% (30)	**<0.001**
Motor functioning impairment (Vineland)	17% (2)	73% (22)	**0.003**
Sleep problems (Tayside)	50% (6)	68% (21)	0.242
Neuropsychiatric liability (CBCL)	0% (0)	55% (17)	0.001*
ADHD liability (CBCL)	0% (0)	16% (5)	0.300*
ODD liability (CBCL)	0% (0)	16% (5)	0.301*
Mood disorder liability (CBCL)	0% (0)	45% (14)	0.003*
Anxiety liability (CBCL)	8% (1)	29% (9)	0.197
Autism liability (SRS)	0% (0)	48% (15)	0.003*

*Note*: Bold number indicates the *p*‐value survives B‐H FDR 0.05 correction for multiple testing.

*p*‐values were derived from logistic regression models were conducted with age and gender as covariates, except for those starred (*). * For some categorical variables where there was 0 cell count for controls, Fisher's exact test was used to derive *p*‐values.

### Association of cognitive, motor and sleep markers with neurodevelopmental and psychiatric outcomes

Within children with 22q11.2DS, behavioural and emotional problems total score (CBCL) was significantly associated with developmental coordination score (*r* = 0.53, *p* = 0.002) and sleep problems (*p* < 0.001) (see Table 5). It should be noted that the behavioural and emotional problems total scores also correlated with global cognitive ability (*r* = 0.38, *p* = 0.037), but this did not survive multiple testing correction. Autistic traits were significantly associated with motor functioning (*r* = 0.63, *p* < 0.001), including both Fine Motor (*r* = 0.54, *p* < 0.002) and Gross Motor (*r* = 0.56, *p* < 0.001). Vineland scales, as well as developmental coordination score (*r* = 0.78, *p* < 0.001). It should be noted that autistic traits were correlated with global cognitive ability (*r* = 0.38, *p* = 0.035), but this did not survive multiple testing correction.

**TABLE 5:** Correlations between developmental traits and neurodevelopmental and psychiatric outcomes.

Developmental trait	Total behavioural & emotional problems (CBCL)		Autistic traits (SRS)	
	*r*	*p*	*r*	*p*
Motor functioning (Vineland)	0.43	0.018	0.63	**<0.001**
Fine motor functioning (Vineland)	0.37	0.043	0.54	**0.002**
Gross motor functioning (Vineland)	0.38	0.034	0.56	**0.001**
Developmental coordination (DCDQ)	0.53	**0.002**	0.78	**<0.001**
Sleep problems (Tayside)	0.60	**<0.001**	0.41	0.023
Global cognitive ability (Mullens)	0.38	0.037	0.38	0.035
Expressive language (Mullens)	0.26	0.15	0.21	0.254
Receptive language (Mullens)	0.36	0.049	0.43	0.017
Fine motor skills (Mullens)	0.32	0.084	0.31	0.088
Visual reception (Mullens)	0.17	0.366	0.19	0.309
Spatial planning (neurocognitive)	0.19	0.303	0.14	0.448
Delay gratification (neurocognitive)	0.23	0.243	0.094	0.636
Stroop task performance (neurocognitive)	0.24	0.198	0.074	0.693
Theory of mind (neurocognitive)	0.00	0.992	0.22	0.369

*Note*: Bold number indicates the *p*‐value survives B‐H FDR 0.05 correction for multiple testing.

### Influence of familial traits on childhood traits

Parental SRS T scores were lower, reflecting lower autistic traits, when compared to those of children with 22q11.2DS; 31 mother‐child pairs, maternal SRS mean = 48.0, 22q11.2DS mean = 60.9, paired *t*‐test *p* < 0.001; 22 father‐child pairs, paternal SRS mean = 49.0, 22q11.2DS mean = 61.1, paired *t*‐test *p* < 0.001. Total SRS score was highly and positively correlated between children with 22q11.2DS and mothers (ICC = 0.47, *p* = 0.046, *n* = 31), whereas there was no significant evidence for an association with paternal SRS score (ICC = 0.28, *p* = 0.22, *n* = 22). Though it should be noted, that fewer fathers were available to complete the SRS compared to mothers.

## DISCUSSION

This study took a genetics‐first approach (Lord & Veenstra‐VanderWeele, 2016) to investigate how early child development is impacted by high genetic risk for neurodevelopmental and psychiatric outcomes. We found that young children with 22q11.2DS show impairments across a broad range of domains, including cognitive, motor, oculomotor, language, social, sleep and neurodevelopmental and psychiatric impairments, relative to non‐carrier sibling controls and population norms. This emphasises that psychiatric liability manifests at an early age, many years before adolescence and young adulthood when psychiatric disorders typically emerge. High levels of phenotypic variability have been previously reported for older children and adults with 22q11.2DS (Chawner et al., 2021; Davies et al., 2020; Jacquemont et al., 2022). Here, we find that phenotypic variability is already present at a very early age. Using cluster analysis, we identified a subgroup of children with 22q11.2DS who exhibited phenotypic profiles indicative of high transdiagnostic psychiatric vulnerability, and a sub‐group of children with 22q11.2DS who exhibited similar development to controls. Furthermore, this provides evidence that a fine‐grained systematic approach to developmental assessment may better inform clinicians of targets for intervention that are tailored to individual children's needs. However, it should be noted that this study is cross‐sectional and exploratory; this work needs replication, and future longitudinal studies are needed to investigate whether the high vulnerability in Cluster 2 does indeed predict greater risk of psychiatric outcomes later in life. More broadly, further longitudinal studies would help identify how the trajectory of vulnerability changes with age, development in some domains may catch up (developmental maturation), whilst other domain deficits may emerge or increase in magnitude with age (developmental lag) or stay constant across childhood (developmental deficit) (Chawner et al., 2017).

We also identified that motor and sleep impairments in early childhood index neurodevelopmental and psychiatric outcomes. We cannot infer direction of effect from this study and these findings are exploratory, but our results highlight motor development and sleep function as early markers of psychiatric risk in children with 22q11.2DS, and mirror findings in older children and adolescents with 22q11.2DS that indicate that sleep and motor impairments index psychiatric risk (Cunningham et al., 2018; Moulding et al., 2020). There are several possible explanations as to why early motor development and sleep problems may index neurodevelopmental and psychiatric risk. First, these traits may directly lead to the development of neurodevelopmental and psychiatric impairment. Another possibility is that motor and sleep problems are a secondary consequence of early neurodevelopmental and psychiatric impairment. Thirdly, motor and sleep function could co‐develop with neurodevelopmental and psychiatric liability, for example, as a consequence of the aberrant brain development seen in 22q11.2DS (Ching et al., 2020; Sun et al., 2020). Our findings for 22q11.2DS corroborate those for polygenic risk for schizophrenia, that is, that genetic risk impacts motor development from an early age many years before the onset of psychosis (Serdarevic et al., 2018).

Our study provides evidence that in addition to the effects of the 22q11.2 deletion, familial traits influence the presence of autistic traits in early childhood in 22q11.2DS. This highlights that factors other than 22q11.2DS predict risk; parental autism scores predicting scores in children with 22q11.2DS could reflect polygenic and other family‐related factors (Jacquemont et al., 2022). Previous studies have found that polygenic scores for schizophrenia and intellectual intelligence were predictive of schizophrenia and intellectual in individuals with 22q11.2DS (Davies et al., 2020).

Previous studies of 22q11.2DS have identified a range of cognitive and psychiatric risk factors in adolescence that predict later development of adult psychiatric outcomes (Antshel et al., 2010; Tang & Gur, 2018). Our work highlights that many of the cognitive and psychopathology risk indicators described in adolescence are disrupted from early childhood. Previous research has indicated that cognitive trajectories of children with 22q11.2DS who later development psychosis start to diverge from age 11 onwards from children with 22q11.2DS who do not develop psychosis (Vorstman et al., 2015). Although our study was not longitudinal and can therefore not make claims about future risk, it is striking that in early childhood it is possible to identify a subgroup (16/30) who appear to be diverging in their development from other children with 22q11.2DS and control siblings, this proportion is in line with the prevalence of psychiatric conditions within school‐age children with 22q11.2DS (54%) (Niarchou et al., 2014). Intervention strategies for children with 22q11.2DS delivered in early childhood may result in greater positive outcomes (Correll et al., 2018). Our study highlights the breadth of early developmental traits disrupted by the 22q11.2 deletion, including gross motor, fine motor, oculomotor, global cognitive ability, a range of specific neurocognitive abilities, social development, measures of neurodevelopmental and psychiatric liability, and sleep function. Our findings indicate the need for young children with 22q11.2DS to receive multidisciplinary support from a range of professionals including paediatricians, physiotherapists, occupational therapists, psychologists, psychiatrists, and early education intervention specialists.

A range of oculomotor impairments have been previously reported in adults with schizophrenia (Gottesman & Gould, 2003; Morita et al., 2020). Exploratory analyses of oculomotor traits in our study found that impairments in smooth pursuit eye movements (*p* = 0.035) and fixation stability (*p* = 0.002) were present in young children with 22q11.2DS. Although our study was cross‐sectional, and the tasks conducted in this study of very young children do not directly map onto tasks administered in previous studies of adults with schizophrenia, our findings indicate that oculomotor differences exist in 22q11.2DS at an age many years before typical age of schizophrenia onset. Our findings highlight support for oculomotor traits as being important potential biomarkers for neurodevelopmental and psychiatric risk, and a major advantage of oculomotor tests for clinical translation is they are non‐invasive and rapid to conduct (Holzman, 2000; Wass et al., 2015).

Although this study had the strength of including a control group, and assessing a broad range of dimensional phenotypes, the findings should be considered in light of a range of limitations. Firstly, children were ascertained for the study on the basis of an existing genetic diagnosis, and this is likely to introduce ascertainment bias towards children with developmental delay as this is a common reason for referral to genetic testing. However, it should be noted, that despite this, considerable variability in outcomes was still seen within this cohort, emphasised by the cluster analysis findings that identified a subgroup of 22q11.2DS children at *lower vulnerability* with similar scores to control siblings. Comparison to population norms and community control data revealed that broadly, the scores of the sibling controls were representative of the general population, but is important to highlight there were a small number of traits whereby sibling controls did differ, highlighting the importance of considering a range of control groups when designing future studies. The differences between children with 22q11.2DS on neuropsychiatric measures could be partly explained by the presence of intellectual disability. In our study we find that although global cognitive ability correlated with autistic traits (*r* = 0.38) and CBCL total scores (*r* = 0.38), and explained 14% of the variance for each outcome, however these findings did not survive multiple testing correction. We cautiously conclude that global cognitive ability may play a role, but cannot fully explain the presence of neuropsychiatric problems in children with 22q11.2DS. This is consistent with findings in older children that find intellectual disability and psychiatric problems are independent consequences of 22q11.2DS (Niarchou et al., 2014). Although the sample size of the study is relatively small, study size is consistent with other early developmental deep phenotyping studies of rare genetic variants (Hogan et al., 2017; Kolesnik et al., 2017; McDonald et al., 2017). We have interpreted findings cautiously, and applied multiple testing corrections, nonetheless our findings should be regarded as exploratory and warrant replication. Our study size was sufficient to detect the large effect sizes conferred by 22q11.2DS on early development, which are likely to have clinical significance, but would not have been powered to detect more subtle differences. The cluster analysis findings should be regarded as exploratory due to sample size and an imbalance in group sample sizes. The transdiagnostic approach of the cluster analysis, as used in previous research (McDougal et al., 2020), was not intended to be used to make broad claims. Rather, the purpose was to investigate whether children with 22q11.2DS and sibling controls clustered together or were distributed across different clusters. Future research is needed in larger samples to investigate the factor structure that underlies the range of cognitive and behavioural impairments present in the early childhood of 22q11.2DS. Large consortium approaches have been applied to investigating the later childhood and adult phenotypes of 22q11.2DS (Chawner, Owen, et al., 2019; Chawner et al., 2021; Schneider et al., 2014; Vorstman et al., 2015), but the same needs to be applied to studies of early development and across a range of risk loci.

## CONCLUSION

22q11.2DS has a large and broad impact on early childhood across motor, social, language and cognitive development, and a range of transdiagnostic clinical risk indicators can be detected from an early age, years before adolescent and adult psychiatric problems may develop. There appears to be a group of young children with 22q11.2DS who diverge from their 22q11.2DS peers and control siblings and express higher levels of neurodevelopmental and psychiatric liability. Motor and sleep function appear to be markers of early neurodevelopmental and psychiatric liability in 22q11.2DS and thus may represent early targets for intervention. Overall, our findings highlight the need for future research that takes a developmental approach to understanding how genetic risk for psychiatric conditions manifests. Large cohorts of children with rare psychiatric variants need to be established from birth and followed longitudinally, complemented by studies that investigate the impact of rare psychiatric risk variants on early development in population cohorts.

## AUTHOR CONTRIBUTIONS

**Samuel J. R. A. Chawner**: Conceptualization; data curation; formal analysis; funding acquisition; investigation; methodology; project administration; resources; visualization; writing – original draft; writing – review & editing. **Amy L. Paine**: Formal analysis; investigation; methodology; resources; software; writing – review & editing. **Matt J. Dunn**: Data curation; formal analysis; investigation; methodology; resources; software; writing – original draft; writing – review & editing. **Alice Walsh**: Data curation; writing – review & editing. **Poppy Sloane**: Data curation; writing – review & editing. **Megan Thomas**: Data curation; writing – review & editing. **Alexandra Evans**: Data curation; formal analysis; writing – review & editing. **Lucinda Hopkins-Jones**: Data Curation; writing – review & editing. **Siske Struik**: Project administration; resources; writing – review & editing. **Jeremy Hall**: Conceptualization, funding acquisition, resources, supervision, writing – review & editing. **Jonathan T. Erichsen**: Conceptualization; funding acquisition; investigation; methodology; resources; software; supervision; writing – original draft; writing – review & editing. **Susan R. Leekam**: Conceptualization; funding acquisition; investigation; methodology; resources; supervision; writing – review & editing. **Michael J. Owen**: Conceptualization; formal analysis; funding acquisition; investigation; methodology; resources; supervision; writing – original draft; writing – review & editing. **Dale Hay**: Conceptualization; funding acquisition; investigation; methodology; resources; supervision; writing – original draft; writing – review & editing. **Marianne B. M. van den Bree**: Conceptualization; formal analysis; funding acquisition; investigation; methodology; resources; supervision; visualization; writing – original draft; writing – review & editing.

## CONFLICT OF INTEREST STATEMENT

MJO, JH and MBMvdB report grants from Takeda Pharmaceuticals, outside the submitted work.

## ETHICAL CONSIDERATIONS

Informed consent was gained from primary carers, and assent from the children. Protocols were approved by the Cardiff University School of Medicine Research Ethics Committee.

## Supporting information

Supporting Information S1

## Data Availability

The data that support the findings of this study are available on request from the corresponding author. The data are not publicly available due to ethical restrictions.

## IMAGINE‐ID Consortium author list

**Table:**

**Surname**	**Initials**	**First name**	**Title**	**Institution**
Raymond	F L	F Lucy	Professor	Department of Medical Genetics, University of Cambridge, UK
Dewhurst	E	Eleanor	Mrs	Department of Medical Genetics, University of Cambridge, UK
Lafont	A	Amy	Ms	Department of Medical Genetics, University of Cambridge, UK
Timur	H	Husniye	Ms	Department of Medical Genetics, University of Cambridge, UK
Wicks	F	Francesca	Mrs	Department of Medical Genetics, University of Cambridge, UK
Ye	Z	Zheng	Dr	Department of Medical Genetics, University of Cambridge, UK
Baker	K	Kate	Dr	Department of Medical Genetics, University of Cambridge, UK
Walker	N	Neil	Dr	Department of Medical Genetics, University of Cambridge, UK
Wallwork	S	Sarah	Ms	Department of Medical Genetics, University of Cambridge, UK
Skuse	D	David	Professor	Great Ormond Street Institute of Child Health, University College London, UK
Denaxas	S	Spiros	Dr	Institute of Health Informatics, University College London, London, UK
Mandy	W	William	Dr	Division of Psychology & Language Sciences, University College London, UK
Wolstencroft	J	Jeanne	Dr	Great Ormond Street Institute of Child Health, University College London, UK
Davies	S	Sarah	Ms	Great Ormond Street Institute of Child Health, University College London, UK
Erwood	M	Marie	Ms	Great Ormond Street Institute of Child Health, University College London, UK
Juj	M	Manoj	Mr	Great Ormond Street Institute of Child Health, University College London, UK
Kerry	E	Eleanor	Ms	Great Ormond Street Institute of Child Health, University College London, UK
Lucock	A	Anna	Ms	Great Ormond Street Institute of Child Health, University College London, UK
Printzlau	F	Frida	Ms	Great Ormond Street Institute of Child Health, University College London, UK
Srinivasan	R	Ramya	Dr	Great Ormond Street Institute of Child Health, University College London, UK
Walker	S	Susan	Dr	Great Ormond Street Institute of Child Health, University College London, UK
Coscini	N	Nadia	Dr	Great Ormond Street Institute of Child Health, University College London, UK
Fatih	N	Nasrtullah	Mr	Great Ormond Street Institute of Child Health, University College London, UK
Denyer	H	Hayley	Ms	Great Ormond Street Institute of Child Health, University College London, UK
Andrews	S	Sophie	Ms	Centre for Neuropsychiatric Genetics and Genomics, Division of Psychological Medicine and Clinical Neurosciences, Cardiff University, UK
Chawner	SJRA	Samuel	Dr	Centre for Neuropsychiatric Genetics and Genomics, Division of Psychological Medicine and Clinical Neurosciences, Cardiff University, UK
Cuthbert	A	Andrew	Dr	Centre for Neuropsychiatric Genetics and Genomics, Division of Psychological Medicine and Clinical Neurosciences, Cardiff University, UK
Challenger	A	Aimee	Ms	Centre for Neuropsychiatric Genetics and Genomics, Division of Psychological Medicine and Clinical Neurosciences, Cardiff University, UK
Hall	J	Jeremy	Professor	Centre for Neuropsychiatric Genetics and Genomics, Division of Psychological Medicine and Clinical Neurosciences, Cardiff University, UK
Lewis	N	Nicola	Ms	Centre for Neuropsychiatric Genetics and Genomics, Division of Psychological Medicine and Clinical Neurosciences, Cardiff University, UK
Owen	MJ	Michael	Professor Sir	Centre for Neuropsychiatric Genetics and Genomics, Division of Psychological Medicine and Clinical Neurosciences, Cardiff University, UK
Ray	S	Sinead	Ms	Centre for Neuropsychiatric Genetics and Genomics, Division of Psychological Medicine and Clinical Neurosciences, Cardiff University, UK
Sopp	M	Matthew	Mr	Centre for Neuropsychiatric Genetics and Genomics, Division of Psychological Medicine and Clinical Neurosciences, Cardiff University, UK
Moss	H	Hayley	Ms	Centre for Neuropsychiatric Genetics and Genomics, Division of Psychological Medicine and Clinical Neurosciences, Cardiff University, UK
van den Bree	MBM	Marianne	Professor	Centre for Neuropsychiatric Genetics and Genomics, Division of Psychological Medicine and Clinical Neurosciences, Cardiff University, UK
Holmans	P	Peter	Professor	Centre for Neuropsychiatric Genetics and Genomics, Division of Psychological Medicine and Clinical Neurosciences, Cardiff University, UK
Bowen	S	Samantha	Ms	Centre for Neuropsychiatric Genetics and Genomics, Division of Psychological Medicine and Clinical Neurosciences, Cardiff University, UK
Bradley	K	Karen	Mrs	Centre for Neuropsychiatric Genetics and Genomics, Division of Psychological Medicine and Clinical Neurosciences, Cardiff University, UK
Birch	B	Philippa	Ms	Centre for Neuropsychiatric Genetics and Genomics, Division of Psychological Medicine and Clinical Neurosciences, Cardiff University, UK
Tong	M	Molly	Ms	Centre for Neuropsychiatric Genetics and Genomics, Division of Psychological Medicine and Clinical Neurosciences, Cardiff University, UK
Ford	T	Tasmin	Professor	Department of Psychiatry, University of Cambridge
Searle	B	Beverly	Dr	Unique Charity, UK
Wynn	S	Sarah	Dr	Unique Charity, UK
Robertson	L	Lisa	Dr	Aberdeen Royal Infirmary Genetics Service
Berg	J	Jonathan	Dr	Ninewells Hospital Dundee Genetics Service
Lampe	A	Anne	Professor	Western General Hospital Edinburgh Genetics Service
Joss	S	Shelagh	Dr	Glasgow Genetics Centre, Glasgow
Brennan	P	Paul	Dr	Northern Genetics Service, Newcastle
Kraus	A	Alison	Dr	Yorkshire Regional Genetics Service ‐ Clinical Genetics
Weber	A	Astrid	Dr	Cheshire & Merseyside Regional Genetic Service
Rawson	M	Myfanwy	Ms	Manchester Centre for Genomic Medicine
Quarrell	O	Oliver	Dr	Sheffield Genetic Services
Vasudevan	P	Pradeep	Dr	Leicestershire Genetics Centre, Leicester
Harrison	R	Rachel	Dr	Nottingham Regional Genetics Service
Williams	D	Denise	Dr	West Midlands Regional Genetics Service, Birmingham
Maher	E	Eamonn	Professor	East Anglian Medical Genetics Service, Cambridge
Kini	U	Usha	Dr	Oxford Genetics Service
Clowes	V	Virginia	Dr	London North West Thames Regional Genetics Service
Van Dijk	F	Fleur	Dr	London North West Thames Regional Genetics Service
Gurasashvilli	J	Jana	Dr	London North East Thames Regional Genetics Service ‐ Great Ormond Street Hospital, London
Mansour	S	Sahar	Dr	London South West Thames Regional Genetics Service, St Georges Hospital, Tooting, London
Holder‐Espinasse	M	Muriel	Dr	London South East Thames Regional Genetics Service Guy's Hospital, London
Watford	A	Amy	Dr	Bristol Clinical Genetics Service, Bristol
Rankin	J	Julia	Dr	Peninsula Genetics Service, Exeter
Baralle	D	Diana	Dr	Wessex Clinical Genetics Service
Procter	A	Annie	Dr	All Wales Regional Genetics Service
