# Supplementary material for: Neurodevelopmental dimensional assessment of young children at high genomic risk of neuropsychiatric conditions
Source: JCPP Adv. 2023 May 4;3(2):e12162. doi: 10.1002/jcv2.12162 (PMC10519742; doi:10.1002/jcv2.12162)
Supplement: Supplementary file 1 — Supporting Information S1 [file JCV2-3-e12162-s001.docx]

**Eye-tracking methodology**

Eye movements were recorded binocularly at 300 Hz using a Tobii TX300 eye tracker (Tobii Technology, Stockholm, Sweden). Participants were seated 62 cm from a 34 × 19 cm screen at a resolution of 1920 × 1080 pixels. The order of oculomotor tasks was pseudorandomised. Before each task, participants completed a short calibration procedure(Stampe, 1993) involving sequential fixation on five targets spanning the range of subsequent task stimuli.

For all oculomotor tasks, a 2° animated ‘morphing animal’ fixation stimulus(Vinuela-Navarro et al., 2017) was shown against a white background. Detail specific to each oculomotor paradigm is outlined in the sections that follow.

Eye-tracking analyses were performed using MATLAB. For all tasks, the eye yielding the most data samples was chosen for analysis. Eyetraces with >40% missing data were excluded from further analysis. Data were cleaned by removal of samples reporting gaze position ≥20% beyond the screen edge or ≥ 10 standard deviations from the median eye position for the entire recording, and gaps in the eye-tracking signal ≤ 25 ms were interpolated by cubic splines. For any remaining gaps in the data, a further 100 ms was deleted either side to remove blink-related artefacts. The data were then filtered with a generalised Savitzky-Golay filter, and eye velocity and acceleration channels were generated (protocol described elsewhere(Dai et al., 2017)). Each resulting eyetrace was then manually inspected, and any showing significant noise, calibration error or other major artefacts were excluded from further analysis. Analysis procedures specific to each paradigm are outlined below.

*Prosaccades*

Prosaccades were assessed in four cardinal directions, each 10° from the screen centre. Each position as tested 16 times, and the order of presentations was pseudorandomised. The four non-central target locations were each cued throughout the task by 2° black squares. Participants were asked to look at the fixation target at all times.

Each trial consisted of the animated fixation target first being shown at the screen centre. When the gaze was detected as being within ±3° of the fixation target, the target jumped to the peripheral location. When the participant had moved their gaze to within ±3° of this new fixation target, a short 'reward’ stimulus was shown (a colourful rotating star and affirmative sound), and the next trial began.

Analysis of prosaccades involved saccade detection using the method described by Engbert and Kliegl(Engbert and Kliegl, 2003). For each trial, the first centrifugal saccade in the cardinal direction of the stimulus was chosen for analysis, so long as that saccade occurred between 100-1000 ms after the target jump and had an amplitude between 50-400% of the amplitude required to land in the correct position. Saccade metrics (latency and landing position error) were separated by horizontal and vertical target displacements, and medians values were calculated across all ‘clean’ trials (those without dropped data) per participant.

*Smooth pursuit*

Horizontal and vertical smooth pursuit were assessed in separate recordings, the order of which was pseudorandomised. For each pursuit axis, the fixation target moved at 10°/s across a 15° range passing through the screen centre, for a total of 40 s. Participants were asked to follow the target at all times.

Analysis of smooth pursuit first involved detection of saccades using established methods(Larsson et al., 2013). Each 15° sweep of the target was considered a trial. For each trial, *gain* (eye velocity with respect to target velocity) was calculated for all non-saccadic portions of the eyetrace, and *pursuit fraction* (the proportion of time spent in pursuit vs saccades) was calculated. Median values for each metric were then calculated across all ‘clean’ trials (those without dropped data) per participant.

*Fixation*

The fixation target was shown at the screen centre, for 30 s. Analysis of fixation involved calculation of the bivariate contour ellipse area for the entire trial(Crossland et al., 2009).

*Data quality control*

The following strict quality control thresholds were applied: data capture by the eye tracker has to be at least 60%, data loss from before and after eye trace processing had to be less than 10%, the eye trace recording had to be at least 30 seconds, and finally all eye traces were visually inspected for significant artefacts which if present were not included in the final dataset. The following table summarises the number of participants before and after applying quality control thresholds.

**Supplementary Table 1:** Number of participants pre and post eye tracking data quality control

**Supplementary Table 2:** Phenotypic contrasts between controls and 22q11.2DS groups taking account of socioeconomic status

This table shows result of a sensitivity analysis, whereby the linear mixed model analysis presented in Table 3 has been repeated including household income (socioeconomic status proxy) as an additional covariate to age and gender.

**Bold underlined** numbers indicate the p-value survives B-H FDR 0.05 correction for multiple testing.

**Supplementary Table 3:** 22q11.2DS and sibling control performance related to population norms and community control data

For traits derived from the Mullen Scales of Early Learning, CBCL, Vineland, and SRS-2 population norms are available and full details can be found in the manuals for these instruments(Mullen, Achenbach, 2001, Sparrow, 2011, Constantino and Gruber, 2012). For the Tayside questionnaire, DCDQ, and Theory of Mind scale, previously published normative data was used (McGreavey et al., 2005, Rihtman et al., 2011, Wellman and Liu, 2004). For the tasks used from the Cardiff Child Development Study(Meeuwsen et al., 2019, Hay et al., 2021, Hay et al., 2014), the raw data from community controls (average age 2.8 years) was accessed from the study investigators; spatial planning n=181, delay gratification n=225, Stroop task performance n=221. Full details of the Cardiff Child Development Study are published elsewhere (Meeuwsen et al., 2019, Hay et al., 2021, Hay et al., 2014). It should be noted for these three tasks, the average age of the community control data was younger which should be factored into interpretation, i.e. comparing children with 22q11.2DS average age 4.1 years to community norm data from children with an average age of 2.8 years. This could explain why sibling controls scored significantly higher than community controls on spatial planning (p<0.001) and the Stroop task performance (p=0.006), and also why children with 22q11.2DS score similar or higher than community norms for spatial planning, delay gratification and Stroop task performance.

Sibling controls scored significantly higher than population norms for internalising problems (p=0.030), social communication (p=0.019).

*for these traits community control data from previous studies were used as population norm data was not available

**Supplementary Table 4:** Correlations between dimensional cognitive and behavioural traits

See Excel table.

**Supplementary Figure 1:** Gap statistic analysis

Gap Statistic analysis identifies the number of K, where the biggest jump in within-cluster distance occurred, based on the overall behavior of uniformly drawn samples. The plot below was generated using the *fviz_gap_stat* function of the *factoextra* R package, and idneitifes 2 clusters as the optimal solution.


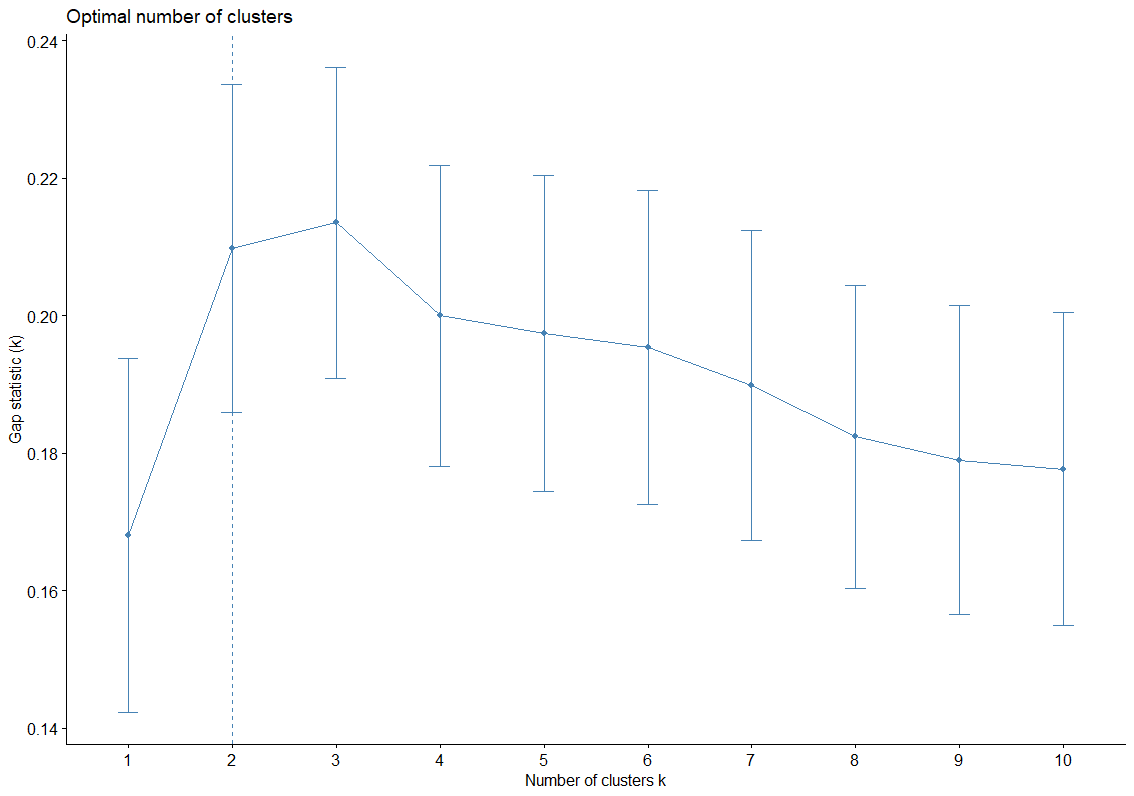


**Supplementary Table 5:** Principal Component loadings

To interpret the principal components presented in Figure 3, the individual loadings were examined. The cut-off of √(1/n) was applied to loadings, with n being the number of traits. This resulted in a cut-off of ±0.2 or higher indicating a significant loading. It should also be noted that loadings for the Transdiagnostic principal component were all positive.

**References**

ACHENBACH, T. (2001). Child Behavior Checklist for Ages 1.5-5 (CBCL/1.5-5). *Reporter,* 10**,** 20.

CONSTANTINO, J. N. & GRUBER, C. P. (2012). *Social responsiveness scale: SRS-2*: Western Psychological Services Torrance, CA.

CROSSLAND, M. D., DUNBAR, H. M. & RUBIN, G. S. (2009). Fixation stability measurement using the MP1 microperimeter. *Retina,* 29**,** 651-656.

DAI, W., SELESNICK, I., RIZZO, J.-R., RUCKER, J. & HUDSON, T. (2017). A nonlinear generalization of the Savitzky-Golay filter and the quantitative analysis of saccades. *Journal of vision,* 17**,** 10-10.

ENGBERT, R. & KLIEGL, R. (2003). Microsaccades uncover the orientation of covert attention. *Vision research,* 43**,** 1035-1045.

HAY, D. F., PAINE, A. L., PERRA, O., COOK, K. V., HASHMI, S., ROBINSON, C., KAIRIS, V. & SLADE, R. (2021). Prosocial and aggressive behavior: a longitudinal study. *Monographs of the society for research in child development,* 86**,** 7-103.

HAY, D. F., WATERS, C. S., PERRA, O., SWIFT, N., KAIRIS, V., PHILLIPS, R., JONES, R., GOODYER, I., HAROLD, G., THAPAR, A. & VAN GOOZEN, S. (2014). Precursors to aggression are evident by 6 months of age. *Developmental science,* 17**,** 471-480.

LARSSON, L., NYSTRÖM, M. & STRIDH, M. (2013). Detection of saccades and postsaccadic oscillations in the presence of smooth pursuit. *IEEE Transactions on biomedical engineering,* 60**,** 2484-2493.

MCGREAVEY, J. A., DONNAN, P. T., PAGLIARI, H. C. & SULLIVAN, F. M. (2005). The Tayside children's sleep questionnaire: a simple tool to evaluate sleep problems in young children. *Child Care Health Dev,* 31**,** 539-544.

MEEUWSEN, M., PERRA, O., VAN GOOZEN, S. H. & HAY, D. F. (2019). Informants’ ratings of activity level in infancy predict ADHD symptoms and diagnoses in childhood. *Development and psychopathology,* 31**,** 1255-1269.

MULLEN, E. Mullen Scales of Early Learning: AGS Edition, 1995. Pearson Assessments: Minneapolis, MN.

RIHTMAN, T., WILSON, B. N. & PARUSH, S. (2011). Development of the Little Developmental Coordination Disorder Questionnaire for preschoolers and preliminary evidence of its psychometric properties in Israel. *Res Dev Disabil,* 32**,** 1378-1387.

SPARROW, S. S. (2011). Vineland adaptive behavior scales. *Encyclopedia of clinical neuropsychology.* (pp. 2618-2621). Springer.

STAMPE, D. M. (1993). Heuristic filtering and reliable calibration methods for video-based pupil-tracking systems. *Behavior Research Methods, Instruments, & Computers,* 25**,** 137-142.

VINUELA-NAVARRO, V., ERICHSEN, J. T., WILLIAMS, C. & WOODHOUSE, J. M. (2017). Effect of stimulus type and motion on smooth pursuit in adults and children. *Optometry and Vision Science,* 94**,** 760-769.

WELLMAN, H. M. & LIU, D. (2004). Scaling of theory‐of‐mind tasks. *Child development,* 75**,** 523-541.
